# Development of a reverse transcription droplet digital PCR (RT-ddPCR) assay for sensitive detection of simian immunodeficiency virus (SIV)

**DOI:** 10.1186/s12985-021-01503-5

**Published:** 2021-02-15

**Authors:** Samuel Long, Brian Berkemeier

**Affiliations:** AIDS and Cancer Virus Program, Frederick National Laboratory for Cancer Research, Frederick, MD 21702 USA

**Keywords:** HIV cure research, SIV, RNA, RT-ddPCR, Raindance, Viral reservoir

## Abstract

**Background:**

Simian immunodeficiency virus (SIV)-infected rhesus macaques constitute an excellent model of human HIV infection. Sensitive detection of SIV RNA in cell and tissue samples from infected animals subjected to treatment regimens becomes especially critical in determining which therapeutic attempts are successful, and consequently, which interventions should be prioritized in HIV cure research.

**Results:**

In this report, we describe the design and testing of a Raindance ddPCR platform-based, sensitive SIV reverse transcription droplet digital PCR (RT-ddPCR) assay by exploring the combinations of various priming conditions and reverse transcriptases, and testing one-step vs. two-step procedures, to eliminate background signal(s) and enable detection and quantification of low level target signals.

**Conclusions:**

Similar reaction conditions and assay validation procedures can be explored for potential development of additional assays for other applications that require sensitive detection of low-level targets in RNA samples.

**Supplementary Information:**

The online version contains supplementary material available at 10.1186/s12985-021-01503-5.

## Background

Quantitative reverse transcription PCR (qRT-PCR) has established itself to be the benchmark for RNA target detection and quantification. The technology yields accurate quantitative data, and significantly simplifies quality control and assay standardization. One of the most prevalent applications of qRT-PCR is for the detection of viral loads [1–3], in which qRT-PCR-enabled data on various causative infectious agents [4] have helped to study disease processes and delineate connections between unique viral sequences and clinical signs and symptoms [5]. Various qRT-PCR assays have been developed to detect and quantify negative-strand RNA viruses (including the measles and mumps viruses, and various viruses that target the respiratory tract) [6, 7], positive-strand RNA viruses (including rhino-, entero- and coronaviruses such as SARS-CoV-2) [8–10], double-stranded RNA viruses (such as human rotaviruses) [11], and retroviruses (HIV, HTLV, and related viruses in animal models, such as simian immunodeficiency virus (SIV)) [12–16]. In most cases, qRT-PCR assays are more sensitive than traditional methods such as viral culture [17], and have led to a significant increase in the accuracy and clinical relevance of patient testing. Several assays have been critical for identifying, isolating and treating patients and defining the viral epidemiology during some of the recent and ongoing pandemics [18, 19].

Despite its tremendous utility, qRT-PCR has several limitations. First, quantitation relies on external reference/standard material, and independent, accurate determination of the reference/standard is critical to successful quantitation by qRT-PCR. Second, extreme sequence heterogeneity exists in some viruses, which can interfere with primer and/or probe binding to target sequences and consequently lead to under-quantification and even non-recognition of targets with significant sequence divergence such as newly identified subtypes and clades. Template sequence heterogeneity also often limits the design of qRT-PCR assays, which requires placement of primer and probe sequences in highly conserved regions. Third, in some diseases, tissue sites have been found to serve as better predictors of disease outcome and indicators of treatment efficacy [20], highlighting the value of sensitive detection of viruses in tissues from infected individuals or animal models. However, nucleic acid extracted from tissues can present challenges during qRT-PCR quantification due to co-purified inhibitors and significant amounts of background nucleic acid (i.e. compared to nucleic acid extracted from plasma), which can both contribute to quantitation inhibition. Consequently, sensitivity limitations of many tissue qRT-PCR assays derive mainly from how much input nucleic acid is allowed in each reaction before inhibition occurs.

Digital PCR conceptually takes a different approach to measure the number of target nucleic acid molecules in a sample. Instead of relying on PCR threshold cycle (Ct) values and standard curves as in real-time PCR (qPCR), digital PCR partitions reactants in each PCR reaction into up to millions of mini-reactions. These reactions are thermocycled to PCR endpoint, the numbers of positive and negative reactions are scored, and the target copy number in the original sample is calculated based on Poisson statistics. Compared to qPCR, digital PCR allows direct absolute quantitation of analyte without the need for external standards or calibration curves, and is therefore not influenced by inaccuracy during reference/standard quantitation. As digital PCR quantitation relies on detecting end point PCR products, this method is less susceptible than Ct-dependent qRT-PCR to inefficient amplification, which can occur due to primer and/or probe mismatches caused by sequence heterogeneity, or inhibitors in samples. Additional advantages include higher quantification precision, especially at lower target template copy numbers, as well as greater multiplexing ability because of digital PCR’s unique amplitude or ratio-based higher order multiplexing [21]. Digital PCR has also been widely used in virus/pathogen analysis [22, 23], in addition to cancer mutation detection [24–27], GMO screening [28], gene and miRNA expression testing, copy number variation (CNV) determination [29–31], as well as nucleic acid reference standard and NGS library quantification [32].

Traditionally, digital PCR presented two limitations compared to qPCR. (1) Limited dynamic range. In digital PCR, dynamic range by definition is determined by partition number that is available for each sample, as ideally each reaction compartment contains at most one target molecule. On chip- and array-based platforms (i.e. Fluidigm BioMark HD, QuantStudio 3D Digital and JN MedSys Clarity and Clarity Plus) partition number per sample is in the range of 10,000 to 45,000, while on a couple of oil emulsion droplet digital (i.e. ddPCR) platforms (i.e. Stilla Naica System and BioRad QX100/200 instruments), partition number per sample is on the order of 20,000 to 30,000. These relatively low partition numbers consequently necessitate dilution of many input samples to achieve accurate measurements [33]. The Raindance ddPCR platform, which is used in the current report, partitions each sample into 10 million droplets (i.e. 6 log dynamic range), making the platform compare favorably to the quantification dynamic range achieved on qPCR systems. (2) Limited nucleic acid input in each reaction. Overloading each reaction with nucleic acids above certain threshold amount in some ddPCR platforms [34] was reported to cause significant droplet deformation, decline of droplet number, and quantitation inhibition (i.e. fewer target-containing droplets reach the required fluorescent intensity at the end of thermocycling). We recently reported [22] that on the Raindance ddPCR platform, at least 8 million mammalian cell equivalent genome can be included in each reaction without causing droplets integrity or numbers to drop. In addition, 4 million mammalian cell equivalent genome was included in each reaction without introducing viral target quantitation inhibition. The Raindance ddPCR platform, therefore, drastically improves the nucleic acid input quantity in each reaction.

Simian immunodeficiency virus (SIV)-infected rhesus macaques constitute an excellent model of human HIV infection [35–41]. Many aspects of infected monkeys such as viral infection, pathogenesis and response to cure strategies closely mirror those of infected humans. Sensitive detection of SIV RNA in cell and tissue samples from infected animals subjected to treatment regimens becomes especially critical in determining which attempts are successful, and consequently, which interventions should be prioritized. In the current report, we describe the development and validation of a SIV RT-ddPCR assay through exploration of combinations of various priming conditions and reverse transcriptases, and testing one-step vs. two-step procedures. The SIV RT-ddPCR assay described here is able to detect low level (i.e. single digit level) viral nucleic acid [22], making it ideally suited for the detection of rare events (such as in the context of antiretroviral therapy in HIV cure studies). Similar reaction conditions and assay validation procedures can be explored for potential development of additional assays for applications that require sensitive detection of low quantity target(s) from RNA samples derived from cell or tissue sources.

## Results

### One-step RT-ddPCR

In the process of developing a ddPCR assay for quantifying SIV RNA, we first tested the option of one-step reverse transcription ddPCR (RT-ddPCR). The “one-step” nomenclature by definition refers to the RT step being performed in the same reaction compartment (i.e. tube, microwell, or droplet) as the PCR step. One-step qRT-PCR has been commonly used in gene quantitation due to advantages such as: (1) Simpler workflow, during which no transfer or procedural manipulation is required once the RT step is initiated, which reduces both risk of contamination and sample-to-sample variability due to reduced number of handling steps. (2) Reduced 3′ and 5′ biases introduced by random oligomers and oligo-dT primers. (3) Single RNA molecules being converted to cDNA without competition and entire cDNA sample being used as template for the PCR step. Both can contribute to enhanced detection sensitivity. (4) Automation potential. The fast and simple procedure allows rapid processing of multiple samples and enables easy automation.

For one-step RT-ddPCR, the SuperScript III One-Step RT-PCR System with Platinum Taq DNA Polymerase was used to perform both the cDNA synthesis and PCR amplification steps. More specifically, the one-step reaction mastermix was combined with the enzyme mix, gene-specific primers, probe(s), test or control RNA sample or reference standard, and droplet stabilizer. The mixture was dropletized, and incubated on a thermocycler with adjustable ramp speed (a slower ramp speed was required for ddPCR as it benefits equilibrating temperature exposure across the droplet population because heat transfers more slowly in emulsified samples than in bulk PCR reaction). The end-point PCR products were then analyzed on the Raindance Sense instrument for droplet counts and fluorescent intensity reading. The data thus generated were in turn analyzed with the RainDrop Analyst software to generate graph and statistical data.

**Fig. 1:**
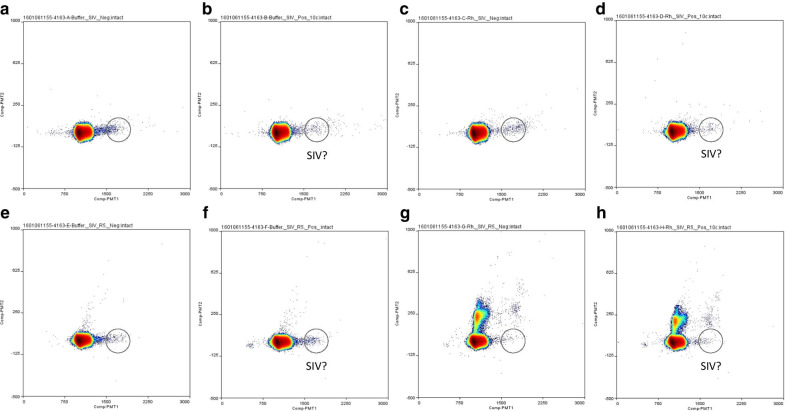
One step RT-ddPCR test. One step RT-ddPCR was performed as described in “Materials and Methods” using the SuperScript III One-Step RT-PCR System with Platinum Taq DNA Polymerase. Assay/sample combinations tested were: SIV single plex assay with buffer (**a**) or SIV RNA standard spike (**b**), SIV single plex assay with 1 μg Rhesus macaque RNA background (**c**) or SIV RNA standard spiked in 1 μg Rhesus macaque RNA background (**d**), SIV and CCR5 duplex assay with buffer (**e**) or SIV RNA standard spike (**f**), and SIV and CCR5 duplex assay with 1 μg Rhesus macaque RNA background (**g**) or with SIV RNA standard spiked in 1 μg Rhesus macaque RNA background (**h**). Detailed experimental conditions are listed in Table 1. Note that in all panels there were background signals in putative SIV target signal region. Quantitation was not done due to background signals

In previous ddPCR tests [22; also see below], assays incorporating minor groove binder (MGB) modified detection probes gave the tightest clusters and clean background in target area (i.e. where positive droplets are located on the plotting space), and were therefore adopted for the PCR stage of the one-step procedure. Four different assay/sample combinations were tested, including SIV assay plus SIV standard spike (Fig. 1a, b), SIV assay plus SIV standard spiked in Rhesus macaque RNA background (Fig. 1c, d), SIV and CCR5 assays plus SIV standard spike (Fig. 1e, f), and SIV and CCR5 assays plus SIV standard spiked in Rhesus macaque RNA background (Fig. 1g, h). One main issue associated with the one-step procedure was background signals in target region when there was no target input, which prevented the utility of the one-step procedure in quantifying low level viruses.

**Table 1:** RT-ddPCR reaction conditions and quantification results

Figure	Procedure	RT enzyme	Priming method	PCR enzyme	SIV input (copies)	SIV count (copies)	PCR thermal cycling condition
1A	One step	SSIII	Gene-specific	Platinum Taq	0	N.D	50 °C 30 min, 94 °C 7 min, 38× (94 °C 14 s, 60 °C 30 s, 68 °C 30 s, 94 °C 1 s), 94 °C 14 s, 60 °C 30 s, 68 °C 5 min 30 s, 98 °C 10 min, 4 °C hold
1B					10	N.D	
1C					0	N.D	
1D					10	N.D	
1E					0	N.D	
1F					10	N.D	
1G					0	N.D	
1H					10	N.D	
2A	Two step	M-MLV	Gene-specific	AmpliTaq Gold	0	0	(RT) 25 °C 15 min; 37 °C 60 min; 90 °C 30 min; 25 °C 30 min; 4 °C hold. (PCR) 95 °C 7 min, 40× (95 °C 15 s, 60 °C 1 min), 98 °C 10 min, 4 °C hold
2B					10	14	
2C		SSIII			0	1	(RT) 25 °C 15 min; 37 °C 60 min; 85 °C 5 min; 25 °C 30 min; 4 °C hold. (PCR) 95 °C 7 min, 40× (95 °C 15 s, 60 °C 1 min), 98 °C 10 min, 4 °C hold
2D					10	14	
2E		M-MLV	Random hexamer		0	0	(RT) 25 °C 15 min; 50 °C 50 min; 90 °C 30 min; 25 °C 30 min; 4 °C hold. (PCR) 95 °C 7 min, 40× (95 °C 15 s, 60 °C 1 min), 98 °C 10 min, 4 °C hold
2F					10	1	
2G		SSIII			0	1	(RT) 25 °C 15 min; 50 °C 50 min; 85 °C 5 min; 25 °C 30 min; 4 °C hold. (PCR) 95 °C 7 min, 40× (95 °C 15 s, 60 °C 1 min), 98 °C 10 min, 4 °C hold
2H					10	2	
3A	Two-step	SSIII	Gene-specific	AmpliTaq Gold	0	N.D	(RT) 25 °C 15 min; 50 °C 50 min; 85 °C 5 min; 25 °C 30 min; 4 °C hold. (PCR) 95 °C 7 min, 40× (95 °C 15 s, 60 °C 1 min), 98 °C 10 min, 4 °C hold
3B					100	N.D	
3C			Random hexamer		0	N.D	
3D					100	N.D	
4A	Two-step	SSIV	Gene-specific	AmpliTaq Gold	0	0	(RT) 25 °C 15 min; 50 °C 10 min; 95 °C 10 min; 25 °C 30 min; 4 °C hold. (PCR) 95 °C 7 min, 40× (95 °C 15 s, 60 °C 1 min), 98 °C 10 min, 4 °C hold
4B					10	10	

Reaction conditions (including the specific procedure applied (i.e. one-step vs. two-step RT-ddPCR), the reverse transcriptase (RT) enzyme used at the RT step, the priming method, the enzyme used at the ddPCR step, and the PCR thermal cycling condition) and quantification results (including SIV input (copies) and count (copies)) are listed. N.D., quantitation not done due to background signals

### Two-step RT-ddPCR

We then tested the two-step RT-ddPCR option. This method separates the RT step and the PCR step in two different reaction vessels. The advantages associated with two-step RT-PCR are: (1) The RT and PCR steps being performed separately, allowing both steps to be optimized to ensure efficient and accurate amplification, i.e. added flexibility regarding choice of primers/priming methods and a wide variety of RT and PCR enzymes. (2) Use of oligo-dT primers or random oligomers for reverse transcription enabling cDNA from a single reverse transcription to be used in several PCRs for analysis of multiple targets, and increased sensitivity for some target templates. (3) Precious RNA samples being transcribed into more stable cDNA without delay for long-term storage and later use.

For two-step RT-ddPCR, a reverse transcriptase combined with a priming method was used to perform the cDNA synthesis step in bulk. The RT enzyme was mixed with other reaction components, including dNTPs, buffer components, random hexamers or gene-specific primer, and test or control RNA sample or reference standard. The mixture was incubated allowing the RT reaction to complete, and then supplemented with components required for the PCR step (including TaqMan genotyping mastermix, primers and MGB-based probe(s)) and the droplet stabilizer. (For the ddPCR step, we previously compared various probe systems and their combinations with different mastermix conditions. MGB probe assays in TaqMan genotyping mastermix, among all the combinations tested, gave the best result in that the signal cluster was tight, there was no background signal in target signal region for no template controls, and ddPCR reads agreed well with input template quantity [22]) This final mixture was dropletized, and incubated in a thermocycler at a ramp speed of 0.5 °C/s. The end-point PCR products were then analyzed on the Raindance Sense instruments for droplet counts and fluorescent intensity reading, and data analyzed with the RainDrop Analyst software to generate graphical and statistical data.

**Fig. 2:**
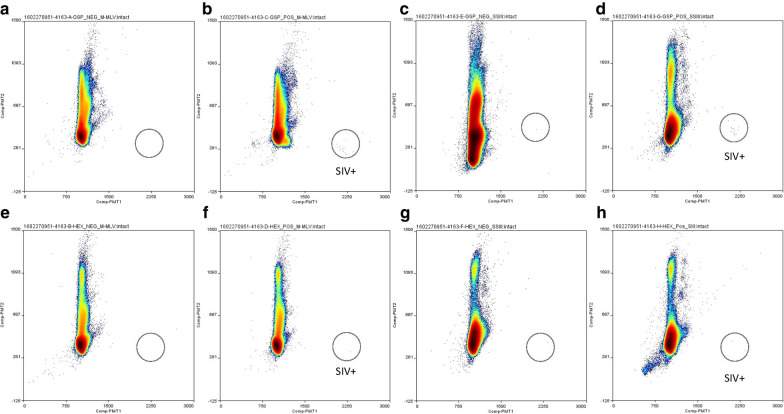
Two step RT-ddPCR test. The following condition combinations were tested during the reverse transcription step of RT-ddPCR: M-MLV RT with gene specific priming (**a** background; **b** SIV RNA standard spike); SSIII RT with gene specific priming (**c** background; **d** SIV RNA standard spike); M-MLV RT with random hexamer priming (**e** background; **f** SIV RNA standard spike); SSIII RT with random hexamer priming (**g** background; **h** SIV RNA standard spike). Each reaction contained 1 μg Rhesus macaque RNA background. The ddPCR step of all reactions was performed with SIV and CCR5 duplex MGB probe assays. Detailed experimental conditions are listed in Table 1

**Fig. 3:**
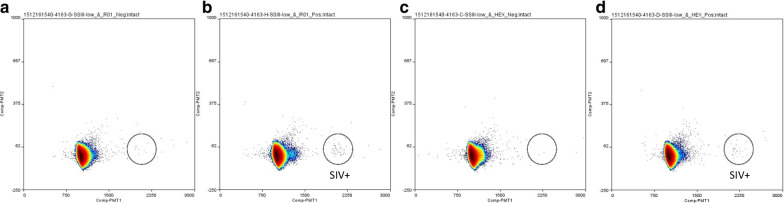
Two step RT-ddPCR test with reduced SSIII RT amount. SSIII RT enzyme was reduced to 20 U in each RT reaction. The condition combinations were: SSIII RT with gene specific priming (**a** buffer background; **b** SIV RNA standard spike); SSIII RT with random hexamer priming (**c** buffer background; **d** SIV RNA standard spike). The ddPCR step of all reactions was performed with MGB probe assays. Detailed experimental conditions are listed in Table 1. Note that in control reactions (**a**, **c**) there were background signals in putative SIV target signal region. Quantitation was not done due to background signals. Random hexamer priming yielded significantly fewer ddPCR signal counts compared to gene-specific priming (compare **d** to **b**)

We tested different cDNA cocktail combinations involving M-MLV and SSIII as RT enzymes, random hexamers and sequence specific-primer as priming methods using a low SIV RNA template input range (i.e. 10 copies of SIV RNA standard), with a major emphasis of identifying conditions that would enable detection of low-levels of viral RNA, typical of those encountered in subjects on prolonged cART suppression. We found that 200U M-MLV and gene specific priming performed well as the target signal background was clean, there was distinct target positive signal cluster, and the ddPCR reading agreed with template input (Fig. 2a, b; Additional File 1: Fig. 1 and Table 1). 200U of SSIII in each RT reaction gave a similar ddPCR readout after the PCR step, however, there was a low background in the SIV target signal region of no template control reactions (Fig. 2c, d). Random hexamers, combined with M-MLV or SSIII (200U in each reaction) yielded significantly fewer ddPCR signal counts compared to template input (Fig. 2e–h), with background signals continuing to be an issue when SSIII was used as the RT enzyme. The background signal issue persisted when the quantity of SSIII was reduced to 20U in each RT reaction, regardless of the priming methods used (Fig. 3).

### Superscript IV (SSIV) test

**Fig. 4:**
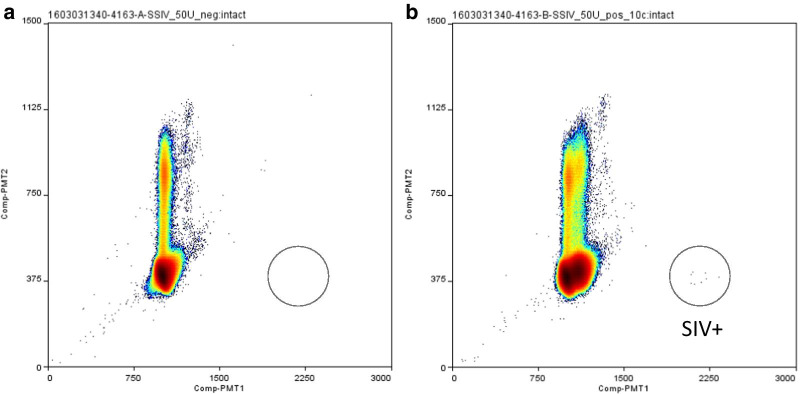
SSIV test. The condition combination was SSIV with gene specific priming (**a** 1 μg Rhesus macaque RNA background; **b** SIV RNA standard spiked in 1 μg Rhesus macaque RNA background). The ddPCR step of both reactions was performed with MGB probe assays. Detailed experimental conditions are listed in Table 1

We proceeded to test another reverse transcriptase, SSIV, in the two-step procedure, as it was previously shown to overcome reaction inhibition from sources such as alcohols, salts, detergents, heparin, hematin, bile salts, formalin, which are typically found in sample preparation reagents, cells and tissues and FFPE samples. This reverse transcriptase can potentially benefit RNA quantification analysis of samples originating from a variety of sources, especially cells and tissues. We showed that SSIV was compatible with the two-step RT-ddPCR protocol and low copy viral signal was successfully identified without background issues when SSIV was used as the reverse transcriptase in the procedure (Fig. 4a, b).

## Discussions

In this report we described the design and testing of a SIV RT-ddPCR assay through testing combinations of different priming conditions, reverse transcriptases, and various procedures, to eliminate background signal(s) and ensure detection and quantification of low-level target signals. We recently reported that the validated assay can detect single digit level SIV RNA in each reaction [22], making it ideally suited for applications involving detection of rare events such as required in many HIV reservoir/cure studies in which viral nucleic acid level is tremendously suppressed. This RT-ddPCR assay was also subjected to additional validation and testing [22]. The linear dynamic range of the assay was at least up to 1 million copies (test upper limit) of viral nucleic acid per reaction [22]. The lower limit of detection for the assay under the M-MLV and gene-specific priming combination condition was determined to be 7 copies per reaction, and the sensitivity and specificity of the assay were determined to be 93% and 100%, respectively.

The reverse transcription step, in which RNA is converted to a DNA template by a reverse transcriptase, can potentially introduce variability and ambiguity through different factors (such as RNA quality and inhibitors) to the final quantitation data in RT-PCR procedures. Endogenous, copurified inhibitors and reagents introduced during sample procurement or extraction often present issues as they can inhibit the reverse transcription step, the PCR step, or both. We recently described using the SuperScript IV reverse transcriptase to overcome severe inhibition at the reverse transcription step. Combining this with Raindance ddPCR system at the PCR step could allow quantifying the SIV viral target in RNA samples that demonstrated more than 99.99% inhibition in qRT-PCR procedure [22]. Therefore, combining a high processivity RT with Raindance ddPCR can potentially expand the repertoire of analyzable tissue RNA samples without the need to remove inhibitors, which is especially important in scenarios where the identity of the inhibitor is unknown.

Different priming methods at the reverse transcription step can lead to different cDNA yield and priming specificity. For example, random priming gives the highest cDNA yield due to the fact that priming initiates from multiple points along the template. However, the majority of cDNA generated through random priming will be from ribosomal RNA and may compete with low level targets. Target/gene-specific priming leads to the most specific cDNA. Consistent with this, we observed that when gene-specific priming was used in the 2-step procedure, there was general agreement between the ddPCR reading and signal input, while random hexamer priming usually yielded significantly fewer ddPCR signal counts compared to template input. It therefore appears that for HIV cure/reservoir studies where the viral detection target signals are at low levels, gene-specific priming should be the preferred priming method during RT-ddPCR analysis.

Quantitation of the SIV RNA reference standard used in this study was based on A260 measurements and the calculated extinction coefficient for the transcript sequence, and validation by terminal dilution qRT-PCR. In Fig. 2b, d, f and h, 10 copies (based on prior quantification) of SIV RNA template were used as input in each reaction. Based on ddPCR results in Fig. 2b, f, the prior quantification under-quantifies the SIV RNA template by about 29% (10 copies vs. 14 copies), assuming the conditions in 2b and 2f allow detecting all target signals present, although another possible contributing factor is the serial dilution step. This highlights the vulnerability of qRT-PCR analysis to inaccuracies introduced during steps such as the quantification and serial dilution of the external calibrator molecules. Nevertheless, valid comparisons can still be made regarding the relative performance of the assays under various RT-ddPCR conditions by comparing the ddPCR signal counts obtained under these conditions.

## Conclusions

Combining gene-specific priming with suitable reverse transcriptases, and using the Raindance ddPCR platform at the signal detection step allowed development of a sensitive SIV RT-ddPCR assay that has many potential applications of interest to HIV reservoir/cure studies. Similar conditions can be explored on the Raindance ddPCR system to enable potential development and validation of additional assays for applications that require sensitive detection of low amount target(s).

## Methods

### RNA extraction and qPCR quantification of SIV viral RNA

RNA isolation and qRT-PCR quantification of tissue-derived SIV RNA followed procedures and conditions as described previously [42–45] and are briefly described here.

Small specimens (i.e. cell pellets or tissue specimens of ≤ 200 mg) were homogenized in 1 mL of TriReagent (Molecular Research Center) in 2 mL homogenization tubes (P000918-LYSK0-A, Bertin Instruments) containing ceramic (zirconium oxide) beads as grinding material using a Precellys tissue homogenizer (Bertin Instruments) according to the reagent manufacturer’s recommendations. Tissue specimens of 200 mg to 2 g quantity range, or 400 mg to 4 g quantity range, were processed in 7 mL (P000935-LYSK0-A, Bertin Instruments) or 15 mL (P000947-LYSK0-A, Bertin Instruments) homogenization tubes containing ceramic (zirconium oxide) beads as grinding material, respectively. For large tissue samples, total RNA was prepared from 1 mL of TriReagent suspension, with residual suspension being archived at − 80 °C for potential additional analysis. All ensuing procedures are based on 1 mL TriReagent homogenate.

RNA isolation from the homogenate included the following steps: (1) Phase separation. The homogenate was stored for 5 min at room temperature to allow complete dissociation of nucleoprotein complexes. The homogenate was spun at 13,000*g* for 1 min, and the top lipid layer removed with a pipette. For phase separation, 0.1 mL 1-bromo-3-chloropropane (BCP) was added to the homogenate. The sample was vortexed vigorously for 15 s. The resulting mixture was incubated at room temperature for 15 min and centrifuged at 14,000*g* for 15 min at 4 °C. Following centrifugation, RNA remained exclusively in the colorless upper aqueous phase. (2) RNA precipitation and wash. The aqueous phase was transferred to a fresh tube containing 240 µg glycogen (Roche 34990920). 0.5 mL of isopropanol was added to the aqueous phase. The mixture was vortexed for 5 s, incubated at room temperature for 5–10 min then centrifuged at 21,000*g* for 10 min at 25 °C. The supernatant was removed and the RNA pellet was washed by vortexing with 0.5 mL of 70% ethanol. The RNA pellet was stored at − 20 °C in ethanol overnight, and was washed a second time with 0.5 mL 70% ethanol. The ethanol wash was then decanted and the RNA pellet allowed to air-dry for 5 min. Recovered RNA was dissolved in 240 µL of 10 mM Tris, pH 8.0 for replicate testing in qRT-PCR protocol.

For qRT-PCR quantification of SIV in tissue-derived RNA, reaction conditions and thermal profiles followed those for the plasma and isolated cell assays as described previously [43, 44] with two exceptions: (1) In the reverse transcription step, the ‘nested’ reverse primer (SIVnestR01) [46], as opposed to random hexamers, was used to prime cDNA synthesis specifically for SIV sequence to avoid generation of non-specific targets and enhance the sensitivity of detection of SIV; (2) 1.25 units of PlatinumTaq (Invitrogen), rather than TaqGold, were used in the amplification steps. For RNA determination, 12 (10 + 2 format) or 6 (5 + 1 format) replicate reactions were tested per sample including a spike of RNA internal control sequence standard [47] (1000 copies per reaction) in two of the 12 reactions (10 + 2 format) or one of the 6 reactions (5 + 1 format) to assess overall amplification efficiency and potential inhibition of the PCR.

### Reverse transcriptases

Three reverse transcriptases were tested in this study. These include: (1) Moloney Murine Leukemia Virus Reverse Transcriptase (M-MLV) (28025013, ThermoFisher Scientific), a recombinant DNA polymerase that lacks DNA endonuclease activity and has a lower RNase H activity. M-MLV has an optimal activity at 37 °C. (2) SuperScript III Reverse Transcriptase (SSIII) (18080093, ThermoFisher Scientific), generated by introducing several mutations into M-MLV to further reduce RNase H activity and increase half-life. SSIII has an optimal activity at 50 °C. Compared to M-MLV, SuperScript III reverse transcriptase was found to produce higher cDNA yields, improved cDNA lengths and enhanced efficiency on GC-rich target RNAs. (3) SuperScript IV Reverse Transcriptase (SSIV) (18090010, ThermoFisher Scientific), an enzyme developed particularly for challenging samples such as poorly purified RNA that contains inhibitors, RNA from formalin-fixed, paraffin-embedded (FFPE) samples, and unpurified RNA. The enzyme demonstrates low variability especially at low amount of input RNA and has the highest thermostability (100% activity up to 56 °C and 90% activity at 60 °C) among the 3 reverse transcriptases.

### One-step RT-ddPCR

For one-step RT-ddPCR, the following RT reaction was prepared in a volume of 50 µL (all concentrations indicate final concentration): 25 µL of 2× reaction buffer and 2 µL of SuperScript III RT/Platinum Taq Mix from the SuperScript III One-Step RT-PCR System with Platinum Taq DNA Polymerase (12574018, ThermoFisher Scientific, Waltham, MA), SIVnestR01 (2 µM), SGag ddPCR forward and reverse primers (600 nM each), SGag ddPCR probe (200 nM) [22, 48], RNA sample, reference standard or buffer, 1xddPCR stabilizer (Raindance) and H2O. For duplex SIV and CCR5 one-step RT-ddPCR, the mixture also contains RCCR5 forward and reverse primers (600 nM each) [22, 48] and RCCR5ProbeMGB (200 nM) [22, 48].

The mixture was then dropletized on Raindance source instrument according to the manufacturer’s instructions. Droplet integrity was monitored by visually examining a portion of the droplets in each lane as they moved through the device during dropletization. Total droplet count data for each sample after dropletization was retrieved from the Source instrument as an independent measure of droplet generation success.

One step RT-ddPCR thermocycling was performed on a Bio-Rad C1000 Touch Thermal Cycler with the following PCR cycling conditions: 50 °C 30 min, 94 °C 7 min (these constitute the RT segments) followed by 38× (94 °C 14 s, 60 °C 30 s, 68 °C 30 s, 94 °C 1 s), 94 °C 14 s, 60 °C 30 s, 68 °C 5 min 30 s, 98 °C 10 min, 4 °C hold.

After thermocycling, droplet fluorescence detection was performed on the RainDrop Sense instrument following the manufacturer’s instruction. At the end of Sense instrument reading, total droplet count data for each sample was retrieved from the Sense instrument.

### Two-step RT-ddPCR

The RT step of the two-step RT-ddPCR was performed in a total volume of 15 µL composed of the following: 5 mM MgCl_2_, 500 nM of each dNTP, 1 mM DTT, 2 µM of SIVNestR01 [46] or 5 µg random hexamers (ThermoFisher), 1× PCR II buffer (ThermoFisher) with 0.2% Tween, 10 U RNaseOUT, M-MLV, SSIII or SSIV (ThermoFisher) reverse transcriptase (amount varies), RNA sample (or reference standard or buffer) and H_2_O. The PCR thermocycling programs for the RT step are: For M-MLV: 25 °C 15 min; 37 °C 60 min; 90 °C 30 min; 25 °C 30 min; 4 °C hold. For SSIII: 25 °C 15 min; 50 °C 50 min; 85 °C 5 min; 25 °C 30 min; 4 °C hold. For SSIV, 25 °C 15 min; 50 °C 10 min; 95 °C 10 min; 25 °C 30 min; 4 °C hold.

After the RT step, the reverse transcription product was directly combined with the following reagents to yield a total mixture volume of 50 µL (the following are final concentrations of reagents in the ddPCR reaction): 1× TaqMan genotyping mastermix, SGag ddPCR forward and reverse primers (600 nM each), SGag ddPCR probe (200 nM), 1× reaction stabilizer (RainDance) and water. In duplex SIV/RCCR5 RT-ddPCR, RCCR5 ddPCR forward and reverse primers (600 nM each) and RCCR5 ddPCR probe (200 nM) were also included in the mixture.

Dropletization was performed on the Raindance sense instrument according to the manufacturer’s instructions and as described above. End-point PCR thermocycling was performed on a Bio-Rad C1000 Touch Thermal Cycler with the following PCR cycling conditions: 95 °C 7 min; 40 cycles of (95 °C, 15 s; 60 °C, 1 min with a ramp rate of 0.5 °C/s); 98 °C 10 min; 4 °C hold. Sense reading and data analysis were performed as described above.

### ddPCR data analysis

Data from Sense runs were analyzed using RainDrop Analyst software to calculate the template copy number by modeling as a Poisson distribution. The formula used for singleplex Poisson modeling was:

$${\text{Copies}}\,{\text{per}}\,{\text{droplet}} = - \ln \left( {1 - p} \right)$$

where p = fraction of positive droplets.

For duplex assay Poisson modeling, the following definition and formula were used:

$$\begin{aligned} & A^{ - } B^{ - } = N \times e^{ - A\% } \times e^{ - B\% } \\ & A^{ + } B^{ + } = N \times \left( {1 - e^{ - A\% } } \right) \times \left( {1 - e^{ - B\% } } \right) \\ & A^{ + } B^{ - } = \, N \times \left( {1 - e^{ - A\% } } \right) \times e^{ - B\% } \\ & A^{ - } B^{ + } = \, N \times e^{ - A\% } \times \left( {1 - e^{ - B\% } } \right) \\ & A\% = - \ln \frac{{1 + \frac{{A^{ - } B^{ + } - A^{ + } B^{ - } }}{N} + \sqrt {\left( {1 + \frac{{A^{ - } B^{ + } - A^{ + } B^{ - } }}{N}} \right)^{2} - \frac{{4A^{ - } B^{ + } }}{N}} }}{2} \\ & B\% = - \ln \frac{{1 + \frac{{A^{ + } B^{ - } - A^{ - } B^{ + } }}{N} + \sqrt {\left( {1 + \frac{{A^{ + } B^{ - } - A^{ - } B^{ + } }}{N}} \right)^{2} - \frac{{4A^{ + } B^{ - } }}{N}} }}{2} \\ \end{aligned}$$

where A^−^B^−^ refers to the number of droplets that contain neither target, A^−^B^+^ refers to the number of droplets that contain target B only, A^+^ B^−^ refers to the number of droplets that contain target A only, and A^+^ B^+^ refers to the number of droplets that contain both targets. N = total number of droplet events.

## Supplementary Information

**Additional file 1. Supplementary Figure 1. Two step RT-ddPCR test with M-MLV RT.** M-MLV RT with gene specific priming condition combination was tested during the reverse transcription step of RT-ddPCR (A: buffer background; B: SIV RNA standard spike). The ddPCR step of both reactions was performed with MGB probe assays. Detailed experimental conditions are listed in Supplementary Table 1. **Supplementary Table 1. RT-ddPCR reaction condition and quantification results.** Reaction conditions (including the specific procedure applied (i.e. two-step RT-ddPCR), the reverse transcriptase (RT) enzyme used at the RT step, the priming method, the enzyme used at the ddPCR step, and the PCR thermal cycling condition) and quantification results (including SIV input (copies) and count (copies)) are listed.

## Data Availability

All data generated or analysed during this study are included in this published article and its supplementary information files.

## Abbreviations

AAALAC: American Association for Accreditation of Laboratory Animal Care
CNV: Copy number variation
Ct: Threshold cycle
ddPCR: Droplet digital PCR
RT: Reverse transcriptase
FFPE: Formalin-fixed, paraffin-embedded
GMO: Genetically modified organism
HIV: Human immunodeficiency virus
HTLV: Human T-cell leukaemia virus
MGB: Minor groove binder
miRNA: MicroRNA
M-MLV: Moloney Murine Leukemia Virus Reverse Transcriptase
NGS: Next generation sequencing
qPCR: Quantitative (real-time) PCR
qRT-PCR: Quantitative reverse transcription PCR
RT-ddPCR: Reverse transcription droplet digital PCR
SARS-CoV-2: Severe acute respiratory syndrome coronavirus 2
SIV: Simian immunodeficiency virus
SSIII: SuperScript III reverse transcriptase
SSIV: SuperScript IV reverse transcriptase
