# Supplementary material for: Development of a reverse transcription droplet digital PCR (RT-ddPCR) assay for sensitive detection of simian immunodeficiency virus (SIV)
Source: Virol J. 2021 Feb 15;18:35. doi: 10.1186/s12985-021-01503-5 (PMC7883996; doi:10.1186/s12985-021-01503-5)
Supplement: Supplementary file 1 — Additional file 1. Supplementary Figure 1. Two step RT-ddPCR test with M-MLV RT. M-MLV RT with gene specific priming condition combination was tested during the reverse transcription step of RT-ddPCR (A: buffer background; B: SIV RNA standard spike). The ddPCR step of both reactions was performed with MGB probe assays. Detailed experimental conditions are listed in Supplementary Table 1. Supplementary Table 1. RT-ddPCR reaction condition and quantification results. Reaction conditions (including the specific procedure applied (i.e. two-step RT-ddPCR), the reverse transcriptase (RT) enzyme used at the RT step, the priming method, the enzyme used at the ddPCR step, and the PCR thermal cycling condition) and quantification results (including SIV input (copies) and count (copies)) are listed. [file 12985_2021_1503_MOESM1_ESM.docx]

**
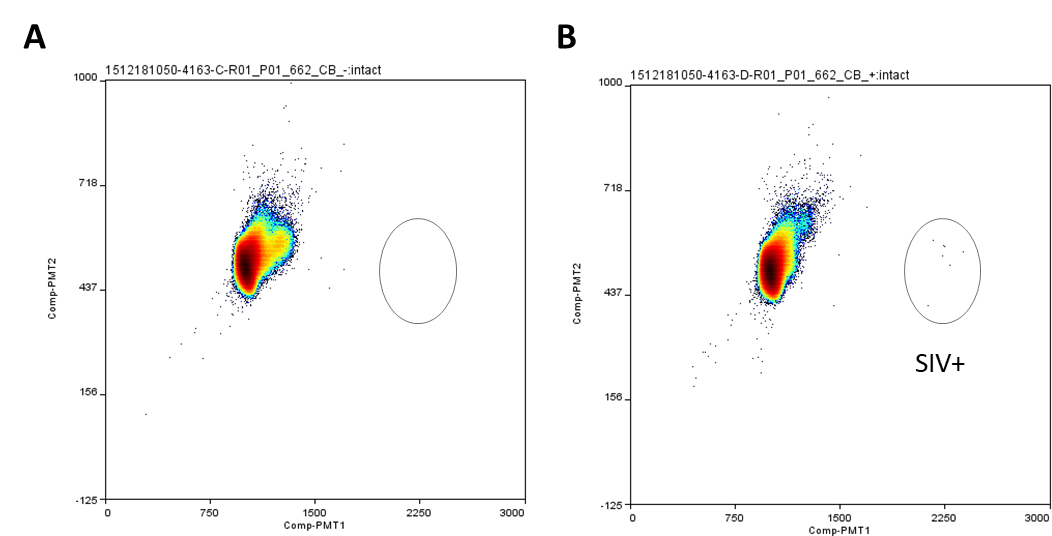
**

**Supplementary Figure 1. Two step RT-ddPCR test with M-MLV RT.** M-MLV RT with gene specific priming condition combination was tested during the reverse transcription step of RT-ddPCR (A: buffer background; B: SIV RNA standard spike). The ddPCR step of both reactions was performed with MGB probe assays. Detailed experimental conditions are listed in Supplementary Table 1.

**Supplementary Table 1. RT-ddPCR reaction condition and quantification results.** Reaction conditions (including the specific procedure applied (i.e. two-step RT-ddPCR), the reverse transcriptase (RT) enzyme used at the RT step, the priming method, the enzyme used at the ddPCR step, and the PCR thermal cycling condition) and quantification results (including SIV input (copies) and count (copies)) are listed.
